# Health Equity Through Black Feminist Healing: A Narrative Review on the Contributions of Black Womxn to Integrative Medicine

**DOI:** 10.1177/27536130251332568

**Published:** 2025-04-08

**Authors:** Eushavia V. Bogan, Elondra D. Harr

**Affiliations:** 1 School of Medicine, University of California, San Francisco, CA, USA; 2 Department of Public Health Education, University of North Carolina at Greensboro, Greensboro, NC, USA

**Keywords:** Black Women, Health Equity, Art, Spirituality, Communal Care, Holistic Care

## Abstract

**Background:**

Black women and Black femme-identifying individuals (referred to as womxn) have developed alternative health practices that support their well-being when navigating oppressive systems. Within the U.S. healthcare system, Black womxn are disproportionally impacted by inequities and discriminatory practices, leading to higher incidences of chronic conditions, limited healthcare access, and higher mortality rates. Integrative medicine has not yet adequately examined or incorporated healing modalities practiced by Black womxn and therefore has not investigated its potential to foster more inclusive care.

**Objectives:**

This critical narrative review aims to explore the contributions of Black womxn to integrative medicine, identify components of Black feminist healing modalities, and discuss future directions for integrating these practices into integrative medicine.

**Methods:**

A critical review was conducted using databases including PubMed, JSTOR, Taylor & Francis Online, and Sage to gather academic and praxis-focused sources. Books and films related to Black womxn healing practices were also examined. Sources were selected based on their focus on non-Western, alternative, and complementary therapies developed and practiced by Black womxn in the United States.

**Results:**

We identified three key categories of Black feminist healing modalities: (1) Communal Care and Communication, which includes practices like storytelling, gossip, and community gathering to foster resilience; (2) Art as a Form of Cultural Strengthening, which emphasizes the use of creative expression for healing and resistance; and (3) Spirituality. These modalities provide tools for Black womxn to resist systemic oppression and promote well-being.

**Conclusion:**

Black feminist healing modalities are crucial for creating inclusive models of care that address the specific health needs of marginalized communities. Incorporating these modalities into healthcare can contribute to health equity by offering culturally relevant and holistic approaches to health for Black womxn and other historically minoritized groups. Future research should focus on developing evidence-based practices for integrating these modalities into clinical settings.

## Introduction

Social inequalities, defined as discriminatory practices and beliefs based on gender, race, sexual orientation, and socioeconomic status, are often exacerbated within health care systems.^1^ It is well documented within the United States (U.S.) that these harmful biases have compromised the well-being of historically marginalized and minoritized communities and have significantly impacted the morbidity, mortality, and livelihood of Black women and other Black femme-identifying people (hereafter womxn^1^). In the U.S., Black womxn have increased incidence of chronic conditions and often face profound barriers to accessing health care. These health disparities and other structural inequities contribute to a shorter life expectancy for Black womxn compared to other demographic groups.^2-4^ Over the past few decades, medical and public health research has begun to reckon with the impact of inequality on people’s mental, physical, and social well-being, however, more needs to be done.

In the U.S., the conceptual framework of integrative medicine emphasizes whole-person and patient-oriented health care^5^ and has the potential to create inclusive models of care for marginalized communities. To provide more holistic healing for patients, the field of integrative medicine has incorporated many non-Western, complementary, and alternative medicines such as Acupuncture, herbal medicine, Yoga, Mindfulness, and other holistic mind-body therapies^6^ into clinical practice. Yet, while the field of integrative medicine incorporates practices of traditional medicines and therapies from Chinese, Indian, Japanese, and a limited number of Indigenous American communities and cultures, there has been less of an emphasis on understanding and utilizing alternative and complementary therapies practiced and developed by Black communities in the U.S.

One of the central principles of integrative medicine is to focus on the mind, body, and soul of patients and provide tools for patients to exercise agency over their health. A study in 2012 demonstrated that racial discrimination was associated with greater complementary and alternative medicine utilization among Black adults as a means to reassert control and self-direction over their health.^7^ This correlation underscores how critical integrative medicine can be for Black well-being and for developing more equitable health practices. However, at this current time, integrative medicine is not fully equipped to serve all communities, and to do so, the field must make a dedicated effort to acknowledge and incorporate radical healing modalities of underrecognized communities, including healing modalities utilized by Black womxn in the U.S. Healing modalities created and practiced by Black womxn are typically grounded in anti-oppressive and emancipatory frameworks, as well as Black feminist theory. Black feminist theory centers on advocating or for empowerment and liberation from all forms of systemic inequality, as well as promoting the well-being of all people.^8^ In the face of systemic oppression, racism, and sexism, Black womxn have channeled their ancestral knowledge, familial practices, and individual ingenuity to create their own mechanisms of care. Yet there is a lack of discourse on the significance of Black feminist healing modalities, which is antithetical to the goals of integrative medicine and is disservice to advancement of equitable health care for Black womxn. This oversight not only perpetuates inequities but also highlights the urgent need for a paradigm shift in how we approach healing and wellness within the field. More work is needed to investigate, incorporate, and uplift Black feminist healing modalities in integrative medicine and the U.S. health care system.

Black feminist healing modalities aim to create accessible forms of care, encourage self-determination, and promote holistic wellness.^9^ This praxis incorporates tools such as storytelling, gossip,^2^ art, poetry, spirituality, herbalism, and other mindfulness techniques to provide ways for Black womxn to practice resistance and resilience to systems that enable structural violence. The U.S. health care system has an extensive history of actions and policies that have caused harm to Black communities. Black communities have faced sexual violence and experimentation to advance medical research, have dealt with discriminatory laws and limited health care access, and have endured the impacts of race-based medicine.^10,11^ This history and ongoing experiences of harm have negatively affected the health and well-being of Black womxn today. Research has documented alarming rates of health disparities in Black womxn communities. Black womxn are disproportionately burdened by STIs and HIV, have higher rates of pregnancy-related morbidity and mortality, are more likely to have hysterectomies than other groups of womxn, have higher rates of cardiovascular disease and anemia, and experience bias from health care clinicians in addition to other health disparities.^12^ However, despite this disproportionate oppression and structural inequality, Black womxn continue to imagine and practice new systems of care that support their survival and liberation. Furthermore, Black feminist healing modalities, as we will explore, uphold the mission and offer tools to improve and sustain health and well-being for all.

Alternative and complementary health practices are deeply valuable to Black womxn due to the need to sustain systems and praxis centering resilience, resistance, survival, and joy when navigating structural and interpersonal oppression and discrimination. It is critically important for integrative medicine to recognize this and appropriately integrate Black feminist healing modalities to uphold its central principle of providing equitable and inclusive whole-person medicine. This paper will explore various Black feminist healing modalities utilized within Black womxn communities and discuss future directions for incorporating these approaches into integrative medicine to improve health equity.

## Methods

We conducted a broad-scope critical review of academic and praxis-focused literature and media to examine the healing modalities utilized by Black womxn throughout the U.S. Critical reviews are nestled under the umbrella of narrative review and provide a flexible framework for synthesizing knowledge from diverse disciplines and allow researchers to draw together disparate ideas, empirical evidence, and theory to envision new ways of conceptualizing data and various issues. This creative approach, however, requires authors go beyond simply synthesizing information and instead play an active role in critically evaluating the relevance and value of sources. In critical review frameworks, authors serve as research instruments and use their unique expertise to appraise and interpret the relevance and value of sources, rather than predominantly describing or summarizing them.^13^ Furthermore, critical reviews do not seek to find definitive answers or solutions to research questions, nor do they attempt to examine all sources on a given topic. Instead, they allow authors to determine sample or theoretical sufficiency to establish the parameters of the search.^14^ This means that result searches end not when a certain number of sources have been identified, but when the results achieve goals and offer meaningful synthesis of the focus.^15^ This approach has played an important role in health profession education and research by advancing understanding of multifaceted topics such as the role of health advocacy in medical training and the impact of supportive doctor-patient interaction on patient care.^16,17^

For our topic of investigation, we used critical review methodology to capture a more comprehensive picture of the vast healing modalities practiced by Black womxn, which included analysis of a mixture of primary sources such as visual art, poetry, ethnography, and fiction as well as secondary sources consisting of journal articles and sociohistorical research. Our synthesis employed the critical review framework and moved iteratively through the following phases: focusing the question, searching for resources, appraising sources, sampling the most impactful sources, and analyzing our results. Our process started with focusing our questions: What are some of healing modalities practiced by Black womxn in the U.S.? Are there any themes or connections between the practices? How can Black womxn healing modalities improve the field of integrative medicine and the quality of care for Black womxn? We then met as a team along with experts (including researchers at the UCSF Osher Center for Integrative Health through the IHEAR Fellowship Program) to develop key search terms and essential sources and data to draw from.

A literature search was conducted using PubMed, JSTOR, Taylor & Francis Online, and Sage journal databases to find articles that focused on healing modalities used by Black womxn. We used keywords including “Black women and Integrative Medicine”, “African American women’s healing”, “Well-being”, and “Holistic approaches to health” to identify sources. From our initial search, further keywords were uncovered including “Art and Healing”, “Black Feminist praxis”, “Black Feminist Healing”, “African Herbalism”, “Black Spirituality”, “Communal Care”, and “Collective Care'' to explore the additional literature related to this topic. We also utilized books and films centered on healing practices used by Black womxn to strengthen our understanding of Black feminist healing modalities from a non-academic perspective.

Our appraisal of the sources was informed by our personal experiences as Black womxn identifying individuals living in the U.S. and with backgrounds working in public health, community health programs, and community organizing. We also applied Black Feminist Thought as a lens for the retrieval, appraisal and sampling of sources. Black Feminist Thought is an epistemology that posits Black womxn possess a unique perspective of their experiences and that their voices should be centralized when theorizing about Black womxn’s lives. Additionally, Black Feminist Thought argues that it is important to not only amplify the discourse of Black intellectuals but also expand research to incorporate knowledge from other disciplines and everyday Black experiences.^18^ To incorporate this lens, our critical review focused on analyzing sources produced by and for Black womxn.

We included texts and media that discussed any integrative, non-Western, or alternative medicines or therapies being developed and used by Black womxn communities. We excluded texts and media that did not focus on complementary or alternative medicines or therapies, did not focus on Black womxn communities, or did not specifically focus on Black womxn living in the U.S. After conducting an iterative process of screening, analyzing, and interpreting text as well as other media, our search yielded a total of 22 texts and media that helped us reach theoretical saturation and identify the following areas: (1) Communal Care and Communication, (2) Art as a form of Cultural Strengthening, and (3) Spirituality as key components of Black feminist healing modalities. Table 1 displays the reviewed sources placed into one of the three categories based on their content.

**Table 1.** List of articles, books, films, and other media That examined Black feminist healing modalities utilized in Black womxn communities in the U.S.

Author(s)	Publication Date	Publication Title	Publication Type	Healing Modalities Discussed
Creator(s)				
botts-ward r.	2023	curating #blackgirlquarantine	Journal article	Communal Care & Communication
Boylorn RM.	2017	Sweetwater: Black Women and Narratives of Resilience	Book	Communal Care & Communication
Collins LG.	2006	Activists Who Yearn for Art that Transforms: Parallels in the Black Arts and Feminist Art Movements in the United States	Journal article	Art as a Form of Cultural Strengthening
Dash J.	1991	Daughters of the Dust	Film	Art as a Form of Cultural Strengthening
Davis S.	2015	Room for Care: Simone Leigh’s free People’s medical clinic	Journal article	Communal Care & Communication
Emezi A.	2018	Freshwater	Book	Spirituality
Farrag H.	2018	The Spirit in Black Lives Matter: new spiritual community in Black radical organizing	Journal article	Spirituality
Grandoit-Šutka A, Flower I.	2021	Mia Birdsong on Freedom, Equity, and Interdependence	Journal article	Communal Care & Communication
Hakim KS.	2022	Care Manual: Dreaming Care into Being	Book	Communal Care & Communication
Huggins E, Shames S, Porter B.	2022	Comrade Sisters: Women of the Black Panther Party	Book	Communal Care & Communication
Imhotep RM.	2022	gossypiin	Book	Communal Care & Communication
Lee ME.	2014	Working the Roots: Over 400 Years of Traditional African American Healing	Book	Spirituality
Machiorlatti JA.	2005	Revisiting Julie Dash’s “Daughters of the Dust”: Black Feminist Narrative and Diasporic Recollection	Journal article	Art as a Form of Cultural Strengthening
Musgrave CF, Allen CE, Allen GJ.	2002	Spirituality and health for women of color	Journal article	Spirituality
Nelson A.	2013	Body and Soul: The Black Panther Party and the Fight Against Medical Discrimination	Book	Communal Care & Communication
Page C, Woodland E.	2023	Healing Justice Lineages: Dreaming at the Crossroads of Liberation, Collective Care, and Safety	Book	Communal Care & Communication
Petteway RJ.	2021	Poetry as Praxis + “Illumination”: Toward an Epistemically Just Health Promotion for Resistance, Healing and (Re)Imagination	Journal article	Art as a Form of Cultural Strengthening
Quaye SJ, Karikari SN, Allen CR, Okello WK, Carter KD.	2019	Strategies for Practicing Self-Care from Racial Battle Fatigue	Journal article	Communal Care & Communication
Rose KM.	2022	The Art & Practice of Spiritual Herbalism: Transform, Heal, & Remember with the Power of Plants and Ancestral Medicine	Book	Spirituality
Shorter-Gooden K.	2004	Multiple Resistance Strategies: How African American Women Cope with Racism and Sexism	Journal article	Communal Care & Communication; Spirituality
Walton QL, Coats JV, Jeffers KS, Blakey JM, Hood AN, Washington T.	2023	Mind, body, and spirit: a constructivist grounded theory study of wellness among middle-class Black women	Journal article	Spirituality
Weems CM, Edwards A, Matsumoto T.	2016	Carrie Mae Weems: Kitchen Table Series	Book	Art as a Form of Cultural Strengthening

## Communal Care and Communication

Communication practices such as storytelling, gossip, and community gathering are central aspects of Black womxn’s healing praxis and provide opportunities for relationship building and collectivism. A key text that demonstrates the healing power of this modality is Robin Boylorn’s *Sweetwater*: *Black Women and Narratives of Resilience*.^19^ Boylorn combines ethnographic storytelling, gossip, poetry, and self-reflections to document the lived experiences of Black womxn in her hometown, the small rural community of Sweetwater, North Carolina. Specifically, she employs narrative inquiry and personal experience to better understand Black womxn’s strategies for making sense and meaning of their lives in the face of social and economic oppression, as well as her life journey as a Black womxn. The resulting ethnography identifies storytelling and gossip as key modalities for Black womxn in Sweetwater to shape and self-determine their lives. She finds that these forms of praxis empowered Black womxn in the community to find the strength to respond to injustices and function as tools to regulate their emotions and improve their well-being. Narratives shared in this text indicate that communication and community relationships are essential to the health and healing of Black womxn.

Ra Malika Imhotep’s book, *gossypiin*, reflects on the role of gossip in solidifying intimate relationships and building shared narratives in Black womxn communities.^20^ Calling upon Black feminist theorists such as Audre Lorde, June Jordan, and Zora Neale Hurston to provide a framework for their analysis, Imhotep unpacks how gossip is a poetic form of knowledge exchange that can be used to document histories and process lived experiences. In the book, Imhotep shares original poetry to retell stories and bits of gossip that explore family history, address sexual trauma, and highlight Black feminist resistance. Additionally, Imhotep uses the process of writing the book to embark on their own healing journey and document the process. *gossypiin* serves as a tangible document of healing and a resource on how gossip can be used to process relationships and lived experiences.

Recent research has started to explore how communication and communal care are used as a form of resilience when facing racism. A qualitative study conducted in 2019 explored how Black educators practice self-care when grappling with racial battle fatigue–including the cumulative psychosocial effects from dealing with racism and discrimination consistently.^21^ Results found that racial battle fatigue is gendered, where cis-gendered women are more likely to experience harsher effects from racial battle fatigue than their male counterparts.^21^ The study also identified that to combat this fatigue, some participants utilized the practice of unplugging from harmful spaces that they must navigate while working in higher education. This process was described as “re-centering” themselves by stepping away from social media and tuning in more with friends and family. In addition to recentering, building connections and creating networks with people who face similar experiences was also highlighted as a strategy. While social science researchers have begun to examine this modality more closely, Black womxn communities have understood the power of communication and connection as a healing modality for many generations. This community knowledge can offer insight on how to foster mindful connections and facilitate more compassionate approaches to care.

An interview with family activist, abolitionist, and futurist Mia Birdsong^22^ unpacks the concept of freedom and the relationship between community and equity using a Black feminist lens. Birdsong attests that communication and reciprocity are essential tools for creating more freedom and stronger interpersonal relationships. By recognizing our interconnectedness to one another and understanding that many community struggles are interlinked, Birdsong outlines how approaching social movements and liberation work through this framework can foster collectivism. Using strategies that center interdependence, and interconnectedness helps promote collaboration and offer opportunities to practice solidarity, which amplifies the power needed to drive systemic change and advocate for liberation. Through recognition of interdependence and practicing collective care, Birdsong affirms that social movements can collaboratively develop a vision of freedom and a culture of care that is inclusive and uplifts all people.^22^

In addition to Birdsong, many Black womxn leaders, activists, and researchers have highlighted the importance of communication in fostering opportunities to feel interconnected and engage in acts of communal care. Communal care is a practice that involves caring for those in your community by utilizing resources to improve the lives of your social network and others around you.^23^ *In Body and Soul*: *The Black Panther Party and the Fight against Medical Discrimination*,^24^ author Alondra Nelson traces the historical roots of communal care within Black American communities and the role of the Black Panther Party in advocating for new modalities of healing. Nelson discusses how the Black Panther Party recognized the crucial link between racial inequality and access to health care, which led them to take measures to address the health care disparities faced by Black communities. In 1968, the Black Panther Party began establishing free health clinics, often called the “People’s Free Medical Clinics,” which provided essential medical services to underserved Black communities in several cities across the United States.^24^ The clinics not only provided clinical care but also engaged with community members to educate them about their health rights, promote wellness through preventive care, and offer programming focused on healthy lifestyle practices. The party’s holistic grassroots approach to health care revealed ways mutual aid and communal care could foster community empowerment, offer education opportunities, and encourage people to participate in advocacy efforts.

*Comrade Sisters*: *Women of the Black Panther Party*, by Ericka Huggins, further delves into the driving forces behind Black Panther Party communal care campaigns and highlights the crucial role that womxn played in community organizing efforts. Huggins provides remarkable stories and photographs of womxn-identifying leaders, organizers, and activists who worked tirelessly, often without recognition, alongside their male counterparts to advocate for Black empowerment, civil rights, and social justice.^25^ These womxn, often referred to as “Comrade Sisters,” were instrumental in challenging societal norms and actively fighting against racial oppression and systemic inequality through community building programs. By documenting their community work and solidary building activities, Huggins demonstrates how Black womxn were central in caring for their community and imagining new ways of being. Their praxis helped envision and enact new social technologies that led to the expansion of welfare programs within the United States.^25^

In *Care Manual*: *Dreaming Care into Being*, Kamra Sadia Hakim meditates on the transformative power of communal care. The manual defines care as an intentional and creative act practiced in nurturing relationships and communities.^26^ They then provide a guide and framework built upon Black feminist and Queer theory to engage in deep introspection on the role community plays in individual healing processes. The manual encourages readers to focus on collective well-being and become more mindful, invested, and interconnected community members. Through this praxis, Hakim champions how communal care can be used to reimagine interpersonal relationships, reconfigure power structures and thus dismantle oppressive systems that harm marginalized people.^26^

## Art as a Form of Cultural Strengthening

Throughout American history, art has been employed as a tool for liberation. It provides a medium for individuals to self-determine their narrative, express feelings, build connections to others, and envision new futures.^27^ Black womxn communities have recognized the potential of art as a healing modality and harnessed its ability to usher in new cultures of care. In Julie Dash’s evocative film *Daughters of the Dust*,^28^ she utilizes filmmaking to explore themes of ancestry, cultural preservation, migration, and the complexities of healing from historical trauma for Black womxn. Dash employs a lyrical and richly visual style, charged with symbolism and poetic imagery to explore characters’ connection to their African roots and the spiritual dimension of their existence.^29^ By interweaving ancestral cosmologies, spiritual practices, and nature-connectedness, Dash’s characters are able to create space for self-compassion, deepen community bonds, and discover pathways for liberation. The film portrays healing as a complex and multifaceted process. It highlights that healing is not just a process of improving physical well-being but also allows for reclaiming and preserving one’s cultural heritage, reconnecting with ancestral practices, and finding a sense of belonging and purpose. The film also recognizes the vital role that womxn play in this healing process. In the film, womxn are depicted as the carriers of culture and traditions and are key to passing down knowledge and wisdom from one generation to the next. *Daughters of the Dust* illustrates how Black womxn can use art to advocate for new systems of healing and uplift Black womxn’s value in communities.

Carrie Mae Weems is another example of an artist employing creative mediums to empower Black womxn communities and highlight the value of their cultures and ways of being. In Weems’ *Kitchen Table Series*, the kitchen table is a symbolic and intimate space where everyday moments, introspections, and interpersonal interactions unfold.^30^ Weems uses these seemingly mundane moments at her table to explore greater themes of race, gender, love, family, and power dynamics. This tableau of different narratives offers a glimpse into Black womxn’s lives, relationships, and experiences as they navigate personal and societal struggles. Her skillful use of lighting, composition, and symbolism elevates everyday scenes into thought-provoking and visually striking representations of the human condition. With these pictures, Carrie Mae Weems invites viewers to contemplate the universality of the human experience while highlighting the unique and often overlooked tenderness and perseverance of Black womxn in contemporary society. The *Kitchen Table Series* offers Black womxn a medium to witness themselves and take pride in their lived experience.

In 2014, Simone Leigh employed creative praxis to develop a transformative art space, *The Free People’s Medical Clinic*.^31^ The installation aimed to center the experiences and needs of Black womxn while also addressing the historical erasure and neglect of their health in the medical system. Drawing inspiration from Afrofuturism and ancient healing practices, the installation incorporates traditional African architecture and contemporary design elements to develop a pop-up clinic that emphasizes the importance of cultural and ancestral knowledge in health care. As a part of the installation, viewers were invited to engage with art and receive different forms of traditional healing, such as massage therapy, dance, reiki, and herbal medicine. Additionally, attendees are offered the *Waiting Room Magazine*, a compilation, edited by Leigh, of fiction, short essays, and images of Black womxn experiences in the health care system.^31^ Together, the multifaceted art installation, along with the magazine, invited visitors to engage with the history of medical mistreatment and discrimination Black womxn face while simultaneously celebrating their resilience and contributions to holistic healing practices and knowledge. Central to *The Free People’s Medical Clinic* is the concept of “care” as an act of resistance and empowerment. By providing a space where Black womxn’s well-being is prioritized, the installation challenges the systemic barriers that have marginalized and silenced their voices within health care. *The Free People’s Medical Clinic* represents a radical departure from conventional medical spaces through the envisionment and development of a safe and inclusive healing environment for Black womxn. Furthermore, it demonstrates how art can be used as a framework for fostering dialogue and activism around Black womxn’s health and well-being.

*Healing Justice Lineages* highlights other critical examples of how art can reduce harm to Black womxn communities.^32^ For example, author Cara Page discusses how the abolitionist organizing project, Changing Frequencies, rooted in Black, queer, indigenous Global South feminism, uses art and storytelling to processes systemic violence and discrimination for Black womxn communities and other historically marginalized peoples. The project curates and produces multimedia installations, immersive experiences, and storytelling interventions focused on healing from the abuse of the medical industrial complex and promoting collective care.^32^ Changing Frequencies has supported the development of activities such as altar building, collective artmaking, and ritual-based events to create spaces for individuals to process trauma and connect to ancestral practices. The organization has also contributed to the creation of a narrative-based history project documenting the evolution of health care within the United States as well as the impact of medical experimentation, scientific racism, racial capitalism, and ableism on the health care system’s practices and policies.^32^ By amplifying stories of both the harm and the resilience strategies of oppressed people, Changing Frequencies serves as evidence of how art-based initiatives can support repair work and create opportunities to envision more equitable and diverse healing practices.

The healing power of art for Black womxn communities is further explored in Petteway’s article, *Poetry as Praxis + “Illumination”*: *Toward an Epistemically Just Health Promotion for Resistance*, *Healing*, *and (Re)Imagination*, which discusses the creative power of poetry as a means to contemplate new healing modalities.^33^ Drawing from Black feminist theory, Petteway interrogates how creating art, particularly poetry, can be used as a health promotion praxis that is responsive and connected to people’s daily realities, especially those of Black communities.^33^ Petteway argues that poetry is a legitimate format for knowledge expression and serves as a testimony of people’s experiences in the world. Poetry offers a direct source of subjective data on people’s lives, which is vital for understanding how to create inclusive systems of care. Furthermore, uplifting the value of poetry can center marginalized voices and thus diversify how we collect data and give narrative control back to the people experiencing oppression. Therefore, poetry as praxis must be valued as a tool to advance integrative medicine, health care research, and health equity initiatives.

In *curating #blackgirlquarantine*, author reelaviolette botts-ward documents her process of weaving together Black feminist theory, art, poetry, and spirit medicine to develop a creative praxis for healing and grief processing.^34^ Through gathering and collaging images of Black womxn and girls who died during the early years of the COVID-19 pandemic and meditating on their lives, botts-ward memorializes their spirits and creates space for connecting with their stories. This artwork was then further expanded through the production of a digital exhibition that incorporates music and multisensory experiences. The exhibition sought to conjure a communal space for emotional release and reflection for Black womxn communities, as well as others in mourning.^34^ Botts-ward’s work corroborates the healing power of art as well as the ways it can inspire the creation of new forms of medicine that can be inclusive of Black womxn healing needs.

## Spirituality

Spirituality and faith-based practices play significant roles in healing for Black womxn and are often used as a resistance strategy to cope with racism, sexism, and oppression. In a qualitative study, Kumea Shorter-Gooden explores various resistance frameworks Black womxn utilize to cope with discriminatory experiences.^35^ Study participants were noted to rely on prayer, their spiritual beliefs, or their relationship with God as a central strategy for coping with stressors. Additionally, the study identifies the concept of “standing on shoulders'', which is the process of acknowledging ancestors and staying connected to one’s heritage as an important tool for promoting emotional and spiritual well-being.^35^ Additionally, the data presented also highlighted how the relationship between ancestry, faith, and spirituality is salient to Black womxn healing practices. Public health implications for spirituality as a healing modality are further discussed by Musgrave et al. in a commentary article analyzing the association between spirituality and positive health outcomes for womxn of color.^36^ The article reviews faith-based program strategies such as The HIV Prevention Faith Initiative of the Centers for Disease Control and Prevention, which utilized gospel artists and music to dispel myths and encourage more awareness around HIV.^36^ This approach exemplifies how public health initiatives can successfully utilize alternative healing practices and partner with spiritual leaders to improve community health and well-being. Moreover, this model demonstrates how utilizing relatable and relevant outreach activities can bolster initiatives focused on Black womxn health. In 2023, another study used grounded theory methodology to survey middle-class Black womxn living in a Midwestern city in the U.S. on what is needed to improve their health and wellness.^37^ Recognizing the immense amount of stress from race and gender-based discrimination experienced by Black womxn, researchers recorded in-depth interviews to analyze first-hand accounts of lived experiences of Black cis-gendered women. The results identified that fostering a connection and balance between mind, body, and spirit was a key aspect of wellness in this population.^37^ Several participants also noted that leaning on Christian principles and stories of Christ provided sources of strength and focus. Anecdotes shared by Black women in the study highlighted how spiritual spaces such as churches or personal time for prayer often served as a tool for processing stress and emotional duress.

Many Black feminist healing modalities embrace connecting to forces larger than the self, that are often not visible, but instead are felt and sometimes supernatural. These practices encourage individuals to create space for contemplating faith, explore community connections to nature, and reflect on ancestral knowledge embedded in people’s psyche. Participating in this form of praxis can offer pathways that can unlock a greater understanding of the self and the ability to build strong connections to others. In Akwaeke Emezi’s novel *Freshwater*, a surreal and semi-autobiographical, coming-of-age story, Emezi examines the concepts of identity, trauma, gender expansion, and the multiplicity of self.^38^ The novel interweaves Igbo cosmology, mythology, and Black feminist theory to unpack the protagonist’s experience with neurodivergence and mental health. In this beautiful work of fiction, Ada, the protagonist grapples with being inhabited by multiple entities known as ogbanje, trickster gods in Igbo religion. Ada’s shared mind and body with the ogbanje serves as a metaphor for the internal struggle and fragmentation individuals can experience when dealing with trauma and the complex emotions it evokes.^38^ The novel recognizes and explores how integrating spirituality and cultural knowledge into care can provide expansive frameworks for processing mental health and trauma.

In *The Art & Practice of Spiritual Herbalism*, Karen M. Rose shares her wisdom on spirituality within Black communities that is deeply connected to the natural world.^39^ Through recipes and anecdotes rooted in Black womxn ancestral knowledge, Rose highlights key plants relevant to the body system and offers comprehensive details on their origins, uses, risks, planetary and energetic qualities, biological composition, historical and mythical applications, and the physiological functions they promote in the body. These recipes can serve as tools for people to utilize in their own homes to support their health and well-being, as well as build energetic connections with plants. Furthermore, Rose interweaves Yoruba cosmology and religion through tales of Orishas into recipes and remedies, to trace ancestral connections to land. These stories about African goddesses tending the Earth strive to inspire Black womxn communities to continue to build spiritual relationships with the plants and nature. In addition to offering healing practices to address the costs of ecological deprivation, systemic oppression, and the generational trauma of enslavement, Rose also explores how plants can offer healing for pain experienced during migration, displacement, spiritual theft, social justice burnout, and emotional labor. By tapping into ancestral knowledge, as well as Black womxn herbal practices, Rose impresses the universal healing power of inherited spiritual technologies, community building, and cultural applications of foods and herbal medicines.

The Black feminist healing modality of building a spiritual connection to the Earth and universe is further discussed in *Working the Roots*: *Exploring American Healing for Over 400 Years* by Michele Elizabeth Lee.^40^ In this book, Lee examines centuries of folk healing traditions and spiritual practices passed down through generations within Black communities, tracing the origins of these healing traditions from their roots in Africa, through the period of enslavement in the United States, and then to the present day. Lee investigates how enslaved Africans’ spirituality and land-based healing practices evolved and adapted over time. The book catalogs the diverse range of healing technologies utilized by Black communities, often by Black womxn healers, including herbal medicine, rootwork, conjuring, and spiritualism. Lee then examines the ways in which these practices often blended elements of African cosmologies, Christianity, and indigenous beliefs to form a potent system of healing.^40^ Through interviews, recipes, and remedies, each chapter celebrates the resourcefulness and ingenuity of Black healers and spiritual practitioners who managed to offer healing within their communities and preserve their knowledge despite facing forced assimilation and oppression.

The power of spirituality as a healing praxis has also been utilized in contemporary social movement building to advocate for equity and collective well-being. Hebah Farrag highlighted the efforts of the Black Lives Matter (BLM) organization to organize various spiritual healing methods and techniques to promote holistic well-being. BLM events have incorporated practices such as prayer to promote resiliency, the creation of altars to remember lives lost due to police brutality, and other spiritually infused methods to heal those impacted by state-sanctioned violence.^41^ BLM organizers have also included other integrative practices such as acupuncture, reiki, therapeutic massage, and plant medicine to promote healing and improve their well-being, as well as to reduce burn-out and heal from grief and trauma.^41^ This inclusive practice suggests that in addition to Black womxn having their own rich, integrative healing modalities, they may also engage in Eastern healing and medicinal practices and other integrative medicines from diverse communities and greatly benefit from inclusive and robust healing modalities.

## Discussion

Black feminist healing modalities are essential for creating more inclusive integrative medicine practices because they provide frameworks and tools to support communities navigating oppression and marginalization. Black womxn have harnessed the healing power of practices such as relationship building, storytelling, creative practice, and spirituality to create holistic medicine and care for themselves and their communities. The praxis of communal care has led to the creation of mutual aid networks, healing spaces, and social support groups for Black womxn in the U.S. Storytelling and community gathering in Black womxn communities have been essential tools for sharing knowledge and providing healing spaces to feel heard and connect with others. Art has offered diverse mediums to process emotions and the intersectionality of Black womxn’s lived experience. It also provides ways to honor the history and culture of the Black and African Diaspora. Similarly, spirituality has helped create personalized healing practices and coping mechanisms for managing emotional and physical stressors experienced by Black womxn and provided ways to strengthen their connection to their culture. These healing modalities grant insight into how Black womxn have cared for themselves and their community, as well as practiced resistance and resilience while facing racism, sexism, and systemic inequality. Furthermore, these praxes present critical knowledge on how integrative medicine in the U.S. can better uplift Black womxn and provide inspiration for how to support other historically minoritized and marginalized groups seeking care.

Findings from the literature support the notion that radical and intersectional healing modalities must be incorporated into care for Black womxn to improve their health and well-being.^42-45^ This is particularly important when considering Black womxn’s agency over their own bodies. There is a long history of Black womxn’s autonomy not being respected by the medical community, as exemplified by Henrietta Lacks, whose cells were used for experiments without permission, and Sara Baartman, who was put on display for white audiences to gaze at her body’s form in the 19th century.^46,47^ This disregard is still present today, as Black trans women and other queer folks are subject to sexual assault and police brutality due to their gender identity, sexual orientation, and race. Black womxn also report experiencing more bias and discrimination in health care.^48,49^ Despite these discriminatory policies and practices, Black womxn have created their own medicine to support their survival and success.

Storytelling, narratives, and other communication practices have been used as radical healing strategies to address issues such as obstetric racism and other forms of racial discrimination, as well as promote safety and equity in health care. Our findings demonstrate that sharing personal narratives, amplifying culturally relevant experiences, and discussing critical issues in Black communities, have empowered individuals to build community and advocate for the wellness of Black womxn. However, the benefits of storytelling and other communication practices are not exclusive to Black womxn communities and can help inform integrative medicine and health care in general of better ways to be inclusive and conscientious of the intersectionality of someone’s lived experience. Recent research has highlighted the ability of a similar practice, called narrative medicine, to offer a greater understanding of patient’s lived experiences and encourage empathy between providers and patients.^50^ Narrative medicine helps generate more complex data, and a deeper understanding of emotional, psychological, and social aspects of people’s health. Research has demonstrated that whole-person medicine is critical for creating policies that improve health outcomes and enable more effective healing services.^51^

In addition to the insights gained through communication practices, other key Black feminist praxis, such as social support, religion and spirituality, and problem-focused coping, have also been recognized as essential for helping Black communities process the emotional and physical impact of racism.^52^ Again, the healing generated from these modalities are necessary for broader societal well-being and individual health. In 2013, a study identified that both communal orientation and investment in the welfare of others were associated with stronger relationship satisfaction, greater daily positive emotions, and greater self-esteem.^53^ The study showed that not only did communal orientation and giving care improve the lives of others, but it also promoted internal well-being and positivity. A systematic review by Balboni et al analyzed more than 15,000 empirical papers between 2000 and 2022 related to the importance of spirituality in serious illness and health. Their research found that not only is spirituality is a social factor associated with health, but also that spirituality is essential in caring for patients with serious illness and spiritual care education is critical for the holistic training of interdisciplinary teams caring for patients with serious illness.^54^ This research was then expanded upon by Long et al, who analyzed how the diversity of spirituality in the U.S. and the incorporation of spirituality as a determinant of health can strengthen U.S. public health care systems and foster more community-centered health care practices.^55^ These findings reiterate the power of spirituality in healing and reinforce the potential of Black feminist spiritual practice to provide holistic care for Black womxn and other historically marginalized communities.

The role of art as a therapeutic tool and praxis for promoting meaningful change has long been recognized by both social science and health care research.^56^ Numerous studies have shown that art is a promising intervention for mental health symptom relief, cancer care, and managing psychosocial distress.^57-59^ Art can promote emotional expression, reduce stress, and create space for imagination and joy. Black womxn have known the healing power of art and used it to reclaim narratives and foster mental resilience in a society where their voices are often marginalized. Despite the growing evidence of the effectiveness of Black feminist modalities and research demonstrating the value of comparable practices, very few of these praxes are being considered or studied within integrative medicine.

Past and current systemic oppression has underscored the need for radical healing modalities to address health disparities due to race, sex, and gender-related trauma. A shift within the field of integrative medicine is needed in order to incorporate Black feminist healing modalities as tools to achieve health equity for marginalized and minoritized groups, particularly for Black womxn. Integrating diverse and inclusive modalities of care within integrative medicine has the potential to revolutionize health care and uplift the significance of Black womxn’s knowledge on how to advance collective well-being. Future research in integrative medicine must consider the importance and relevance of Black feminist healing modalities to foster more equitable health care and study how to integrate these practices in thoughtful and empowering ways.

## Limitations

This review has potential limitations. Findings from this review are broad, as narrative reviews are based on the author’s interpretations and do not follow a strict protocol for study selection and data extraction. This may affect the objectivity of the review process. We attempted to mitigate this by conducting multiple cycles of screening, analyzing, and interpreting findings from the literature based on our guiding research question to examine Black feminist healing modalities being used in Black womxn communities in the U.S. While this narrative review offers valuable insights and synthesis of the existing literature on Black feminist healing modalities, future research should aim to address these limitations by conducting more rigorous systematic reviews which includes more diverse sources of evidence and findings from the field. Additionally, little research focuses on examining Black feminist healing modalities in the context of integrative medicine. More research is needed to better understand how Black feminist healing modalities are used within Black womxn communities to promote health and well-being.

## Call to Action

Clinicians, researchers, and other health professionals within integrative medicine must reflect on their current practice sand make a concerted effort to gain the trust of the communities they serve, including Black womxn. To build trust, health professionals need to educate themselves on the systems and policies that inform health and adopt practices that uplift and value historically marginalized and minoritized communities’ knowledge and skills to improve equity and public health. Professionals within integrative medicine must seek to understand different cultures, perspectives, and healing practices when creating prevention and treatment strategies. Black feminist healing modalities are essential practices that can enhance the well-being of Black womxn and society as a whole. They also invite us to imagine and create new whole-person centered and community-based health care for all.

Integrative medicine should approach the knowledge and wisdom inherent in Black feminist healing culture and praxis with humility. Acknowledging that healing modalities utilized by Black womxn may not be conceptually new, however, it is important to emphasize greater understand of the specific cultural values and meaning of these practices. Integrative medicine holds the potential to help bridge Western medicine with ancestral knowledge and provide care that is representative and relevant to people’s varied experiences. Therefore, clinicians, researchers, and other professionals within the field must look to Black history, art, praxis, spiritual practice, and cultural traditions to develop integrative medicine programs tailored to center Black womxn’s voices and meet their needs. Furthermore, Black feminist healing modalities should be recognized and included as viable options for people looking to utilize holistic, community-based healing practices in addition to biomedical care and treatment. In recent years, there has been a call to increase the number of Black health professionals in the field and increase the amount of community-engaged research in BIPOC communities. In addition to that goal, we assert that more research is needed to support the establishment of evidence-based practice of Black feminist healing modalities within integrative medicine.

## Notes

1. In this article we use the term, womxn, to highlight and be inclusive of the many genders who have lived the experience of being a Black womxn or girl. This definition is borrowed from *Black Feminist Future*. Black Feminist Future. Black Womxn Are Essential. Published online November 10, 2022. Accessed September 30, 2024. https://blackfeministfuture.org/black-womxn-are-essential/.

2. The term ‘gossip’ refers to using interpersonal communication to foster social connection and establish a shared understanding of social and cultural norms within an individual’s social network.

Jolly E, Chang LJ. Gossip drives vicarious learning and facilitates social connection. *Curr Biol*. 2021;31 (12):2539-2549.e6. doi:10.1016/j.cub.2021.03.090.
